# Instructional Autonomy, Teacher Self-Efficacy, and Reported Emphasis on Students’ Social and Emotional Skills Development: A Design-Sensitive Analysis of Mathematics Teachers in TALIS 2024 Türkiye

**DOI:** 10.3390/bs16091601

**Published:** 2026-09-09

**Authors:** Ramazan Erol, Atilla Özdemir

**Affiliations:** 1 Department of Mathematics and Science Education, Faculty of Education, Afyon Kocatepe University, 03200 Afyonkarahisar, Türkiye; 2 Department of Mathematics and Science Education, Faculty of Education, Süleyman Demirel University, 32260 Isparta, Türkiye; atillaozdemir@sdu.edu.tr

**Keywords:** TALIS 2024, instructional autonomy, teacher self-efficacy, social and emotional learning, mathematics teachers, mediation analysis, complex survey design

## Abstract

Social and emotional skills can be addressed within ordinary mathematics activities, but the teacher-level conditions associated with such emphasis remain unclear. This study examined whether mathematics teachers’ perceived instructional autonomy was associated with their reported emphasis on students’ social and emotional skill development and whether overall teacher self-efficacy statistically carried part of that association. Data came from 863 teachers in 352 schools in TALIS 2024 Türkiye. Analyses retained the teacher weight, school clustering, and the 100 official Fay-BRR replicate weights for the separately calibrated ISCED 2 and ISCED 3 populations. Instructional autonomy was positively associated with self-efficacy and with reported social–emotional emphasis, and self-efficacy was positively associated with the outcome. The statistical indirect association was positive (B = 0.140, 95% CI [0.100, 0.179]); all four primary hypotheses were supported. The association was positive at both educational levels, and no statistically reliable difference from teachers of other subjects was detected. The findings identify a design-sensitive association pattern in a substantively meaningful mathematics-teaching context, but they do not establish temporal or causal mediation and should not be interpreted as evidence about observed practice or student outcomes.

## 1. Introduction

Social and emotional learning (SEL) concerns capacities that support the regulation of thoughts, emotions, and behaviour, understanding of others, constructive relationships, and responsible decision-making (Collaborative for Academic, Social, and Emotional Learning [CASEL], 2020). School-based intervention research associates systematic attention to these capacities with social–emotional, behavioural, and academic outcomes, while also showing that implementation conditions matter (Durlak et al., 2011). In subject classrooms, however, teachers may address such capacities through everyday instructional decisions rather than through a separate SEL programme.

TALIS 2024 provides a teacher-reported indicator of this instructional emphasis. T4SESDEV records how frequently teachers report working in a target class on students’ management of emotions, thoughts, or behaviour; empathy; healthy relationships; and caring and constructive choices (OECD, 2025a, 2025c). It is not a measure of teachers’ own social–emotional competence, an observed intervention, enacted classroom practice, or student outcomes. Throughout this article, it is therefore interpreted narrowly as teachers’ reported emphasis on students’ social and emotional skill development.

The study focuses on mathematics teachers because mathematical activity provides a substantively meaningful context for examining such emphasis. Students may need to persist when a solution is not immediately available, regulate frustration and uncertainty, respond constructively to errors, explain their reasoning, consider alternative strategies, and participate in collaborative problem solving. At the same time, TALIS measures domain-general areas, some of which map less directly onto mathematics instruction. The mathematics focus therefore establishes the instructional context of the analysis; it does not presume that the estimated relationships are unique to mathematics.

The focal question is whether perceived instructional autonomy, overall teacher self-efficacy, and reported social–emotional emphasis are statistically interconnected. Prior research links autonomy with self-efficacy (De Neve et al., 2015; Gong, 2025; Peng et al., 2022; Wang et al., 2026) and self-efficacy with reported instructional practices and classroom processes (Altun et al., 2025; Yoon & Goddard, 2025; Zee & Koomen, 2016; Zhang et al., 2025). Recent TALIS 2024 work has also begun to examine organisational and relational antecedents of teachers’ SEL-related practices (Qian et al., 2026). Yet these studies have rarely been integrated around the autonomy–self-efficacy–reported emphasis structure, which has not been examined among mathematics teachers in the Türkiye sample. This study addresses that gap while preserving the complex sampling design and distinguishing primary hypotheses from supplementary robustness analyses.

### 1.1. Social–Emotional Skill Development Within Mathematics Instruction

Within an ordinary mathematics lesson, emphasis on social and emotional development need not involve separate activities devoted to emotions or behaviour. Emotion and behaviour management may be implicated when students sustain effort during productive struggle or productive failure, respond to mistakes and feedback, and manage the uncertainty of an unresolved task (Hiebert & Grouws, 2007; Kapur, 2008). These demands are especially relevant in a subject where negative affect and mathematics anxiety are well documented (Ashcraft & Kirk, 2001; Ramirez et al., 2018) and where mathematical resilience describes a stance of continued engagement despite difficulty (Johnston-Wilder & Lee, 2010).

The social dimensions can likewise be embedded in mathematical activity. Sociomathematical norms regulate what counts as an acceptable explanation, a different solution, or a productive disagreement (Yackel & Cobb, 1996). Listening to a peer’s partially correct reasoning, building on an alternative strategy, distributing participation, and critiquing an argument rather than its author provide concrete opportunities for perspective-taking and constructive relationships. Supportive feedback, normalisation of errors, structured participation, and cooperative interaction can therefore be integrated into mathematics instruction rather than added externally (Molyneux & Diamond, 2025).

The correspondence is nevertheless uneven. Persistence, regulation of frustration, responses to mistakes, and participation in mathematical discussion have a direct pedagogical connection to mathematics learning. Broader T4SESDEV content such as empathy, healthy relationships, and caring choices is linked more indirectly through classroom interaction norms. The scale should consequently be understood as a domain-general teacher report administered to mathematics teachers, not as a mathematics-specific SEL measure. The latter subject comparison tests whether the focal coefficients differ detectably from those of teachers of other subjects; it does not determine whether the same skills carry identical pedagogical meanings across subjects.

### 1.2. Instructional Autonomy, Teacher Self-Efficacy, and Reported Emphasis

T4AUTCH represents teachers’ perceived discretion in the target class over flexible curriculum implementation, teaching methods, assessment activities, and lesson design (OECD, 2025a, 2025c). This teacher-level instructional autonomy differs from autonomy-supportive teaching directed toward students’ volition (Reeve & Cheon, 2021). It defines a perceived decision space but does not determine how that latitude is used. TALIS research nevertheless associates professional discretion with adaptive and innovative teaching-related behaviour (Lin, 2022; Nguyen et al., 2021).

Instructional autonomy may be consequential because it widens the range of choices that teachers perceive as legitimately available. Discretion over task selection, assessment, lesson design, and flexible curriculum implementation can create opportunities to adapt instruction to students’ responses and to establish interaction norms that support persistence, explanation, and collaboration. Yet autonomy remains a necessary-but-insufficient condition: the same latitude can be used for content coverage, examination preparation, differentiation, or relationship building. The analysis therefore asks how perceived discretion is associated with reported priorities rather than assuming that autonomy has a uniform instructional expression.

Within social cognitive theory, self-efficacy concerns beliefs about one’s capability to organise and execute courses of action (Bandura, 1986, 1997). Teacher self-efficacy spans student engagement, instruction, and classroom management (Tschannen-Moran & Woolfolk Hoy, 2001); TALIS combines corresponding domains into T4SELF. Autonomy may provide opportunities for efficacy-relevant mastery experience, although this premise cannot be observed directly here and reciprocal ordering remains plausible (Pfitzner-Eden, 2016). Stronger capability beliefs may, in turn, be associated with teachers’ willingness to engage students, organise interaction, and manage the interpersonal demands involved in reported social–emotional emphasis. The prosocial classroom model similarly positions teacher characteristics and professional functioning as proximal to classroom social–emotional conditions (Jennings & Greenberg, 2009).

Social cognitive theory further specifies why autonomy and capability beliefs may be related. Enactive mastery is a principal source of efficacy information, alongside vicarious experience, social persuasion, and physiological or affective states (Bandura, 1997). When teachers can initiate instructional choices and evaluate their consequences, they may accumulate experiences that are interpreted as evidence of their capability. This mechanism is plausible but not observed in TALIS: the survey does not record prior mastery experiences, and teachers with stronger efficacy beliefs may also seek, negotiate, or perceive greater discretion (Pfitzner-Eden, 2016).

Empirical findings are consistent with a positive autonomy–efficacy association. De Neve et al. (2015) linked autonomy with beginning teachers’ self-efficacy and differentiated-instruction practice, while Peng et al. (2022) reported a positive autonomy–teaching efficacy relationship. TALIS-based studies likewise locate autonomy and self-efficacy within broader models of innovation and organisational commitment (Gong, 2025; Wang et al., 2026). These studies support the proposed association but do not resolve its direction, particularly because professional discretion and efficacy beliefs may be reciprocally related.

The second component of the model is supported by evidence connecting teacher self-efficacy with engagement, instructional support, classroom organisation, and teacher–student interaction (Zee & Koomen, 2016). TALIS studies similarly associate self-efficacy with reported instructional practice and place it between professional conditions and instructional outcomes (Altun et al., 2025; Yoon & Goddard, 2025; Zhang et al., 2025). For the present outcome, capability beliefs may be especially relevant because attending to students’ emotion regulation, participation, relationships, and constructive choices can require the simultaneous organisation of instruction, engagement of reluctant students, and management of interpersonal processes. These links concern reported teaching priorities, not observed instructional quality.

These arguments imply a statistical indirect-association structure: perceived instructional autonomy is associated with overall self-efficacy, which is associated with reported social–emotional emphasis, while autonomy may retain a direct association with that emphasis. The term mediation describes the model decomposition, not an identified causal process. All focal variables were measured once by the same teacher, with reference to the same target class. Shared method variance is therefore a specific threat to the self-efficacy-to-outcome path and the indirect association (Jerrim et al., 2025; Podsakoff et al., 2003), and temporal precedence, reverse ordering, and unmeasured common causes cannot be excluded (VanderWeele, 2016).

A positive direct association is also plausible. Greater discretion may allow teachers to incorporate social–emotional attention into task design, feedback, assessment, and classroom interaction even after accounting for overall self-efficacy. This path is less directly established in the literature than the autonomy–efficacy and efficacy–practice associations. H3 therefore concerns the conditional direct association c′, whereas the total association c is reported as a separate descriptive quantity; neither is interpreted as a causal effect.

### 1.3. Study Contribution, Hypotheses, and Extensions

The Türkiye context adds substantive relevance: teachers work within nationally specified curricular expectations while social and emotional development has gained prominence in curricular reform (Alemdar & Aşıkcan, 2026; OECD, 2025b). The study’s primary contribution is to estimate a focused autonomy–self-efficacy–reported emphasis structure among mathematics teachers while retaining teacher weights, school clustering, and the separate TALIS target-population structure. Mathematics is treated as a pedagogically meaningful context, not as a context in which the relationships are assumed to be statistically distinctive.

The study’s contribution is not the statistical mediation procedure itself. It lies in joining three elements that are usually examined separately: perceived instructional discretion, a broad index of professional capability, and a new TALIS 2024 indicator of reported social–emotional emphasis. The H5 extension also addresses a specificity concern. T4COMSEL is closer in content to the outcome than T4SELF, but it measures comfort rather than a capability judgement. The analysis can therefore test whether broad capability and an SEL-specific disposition remain nonredundant correlates; it cannot adjudicate whether a domain-matched efficacy belief would supersede a general one (Bandura, 1997).

A second contribution is methodological. Türkiye’s ISCED 2 and ISCED 3 samples are separately calibrated TALIS target populations, so the pooled estimate is presented as an investigator-defined weighted union and is accompanied by full level-specific results. The analysis also retains official replicate-weight inference, separates confirmatory H1–H4 tests from unpreregistered extensions, and interprets T4SESDEV as reported instructional emphasis rather than observed practice. These decisions limit the scope of the claims while making the estimand and its uncertainty explicit.

**H1.**  *Perceived instructional autonomy is positively associated with overall teacher self-efficacy.*

**H2.**  *Overall teacher self-efficacy is positively associated with teachers’ reported emphasis on students’ social and emotional skill development.*

**H3.**  *Perceived instructional autonomy is positively associated with teachers’ reported emphasis on students’ social and emotional skill development, conditional on overall teacher self-efficacy (direct association c′).*

**H4.**  *Overall teacher self-efficacy carries a positive statistical indirect association between perceived instructional autonomy and teachers’ reported emphasis on students’ social and emotional skill development.*

**Figure 1:**
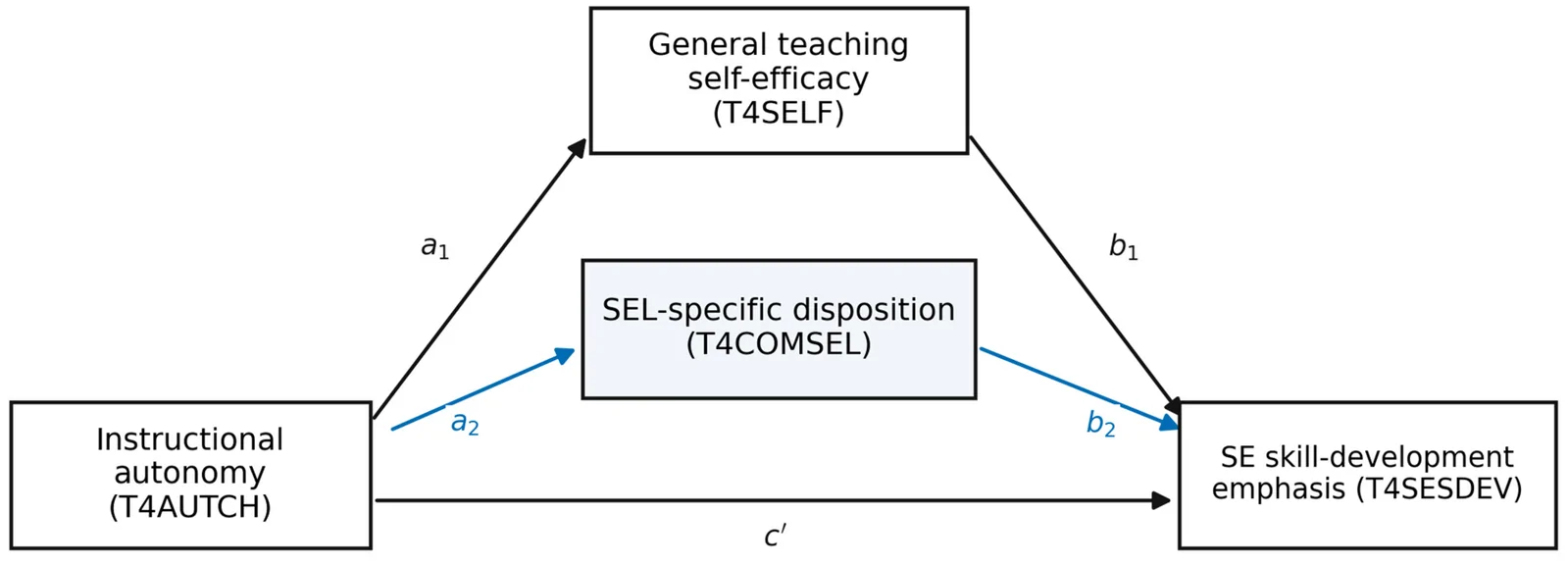
Conceptual model. Note: X = T4AUTCH; M = T4SELF; Y = T4SESDEV. In the figure, a_1_ and b_1_ denote the primary model paths (H1 and H2), a_2_ and b_2_ the corresponding paths of the parallel extension (H5), and c′ the direct association conditional on the mediator (H3). The primary indirect association is a_1_ × b_1_; the decomposition is algebraically equivalent to a simple regression-based mediation specification (Hayes, 2022). H5 adds T4COMSEL as a secondary parallel extension. Arrows denote hypothesised statistical associations and do not imply causal effects.

Two secondary analyses examine the scope of the primary finding. TALIS 2024 includes T4COMSEL, teachers’ comfort with the social and emotional aspects of teaching. Because comfort is a disposition rather than a capability judgement, it is not treated as a domain-matched efficacy measure. H5 asks whether overall self-efficacy and this SEL-specific disposition retain unique associations when modelled together. An exploratory sensitivity check asks whether growth mindset conditions the autonomy-to-efficacy association. These extensions are reported as supplementary to H1–H4 rather than as co-equal contributions (Figure 1).

**H5**  **(extension).** *Overall teacher self-efficacy and the SEL-specific disposition each carry a positive indirect association and retain a unique association with the outcome when modelled simultaneously.*

**RQ1**  **(sensitivity check).** *Does a teacher’s growth mindset condition the association between instructional autonomy and overall teacher self-efficacy, and consequently the indirect association?*

## 2. Materials and Methods

### 2.1. Data Source, Target Populations, and Analytic Sample

This secondary analysis used the Türkiye component of TALIS 2024, a cross-sectional survey with a stratified two-stage probability design in which schools were selected first, and eligible teachers were sampled within schools (OECD, 2025a, 2025c). Türkiye participated at lower secondary (ISCED 2) and upper secondary (ISCED 3). TALIS treats these levels as separate target populations with separately calibrated final and replicate weights.

The primary pooled specification therefore represents an investigator-defined weighted union of the two mathematics teacher populations rather than an official OECD population. Level-specific estimates are reported alongside it and given equal interpretive status. Mathematics teachers were identified from the main subject taught in the TALIS target class. The eligible dataset contained 883 teachers in 357 schools; complete data on the focal variables, covariates, weight, and school identifier yielded 863 teachers in 352 schools (ISCED 2: 468 teachers in 194 schools; ISCED 3: 395 teachers in 158 schools). Item-level missingness was at most 1.02%, and the eligible-to-analytic reduction was 2.3%; available-case comparisons are reported in Supplementary Table S1.

The target-class module was administered on questionnaire forms 2 and 3, representing approximately two-thirds of the teacher weight at each level. Because form assignment was random but the final teacher weight was not recalibrated for form, analytic weight sums describe the composition of the analysed union and should not be interpreted as totals for the full teacher population. All substantive models were teacher-level models. The official weights and replicate weights addressed unequal selection and design-based precision; school-cluster estimators were retained only as auxiliary comparisons.

The two levels contributed comparably to the pooled analysis: ISCED 2 represented 55.7% and ISCED 3 represented 44.3% of the summed analytic teacher weight. Forms 2 and 3 represented 66.5% of the teacher weight at ISCED 2 and 66.4% at ISCED 3; valid target-class subject information represented 65.5% and 64.6%, respectively. The near-identical form coverage reduces concern that the relative level composition was distorted, but the unrecalibrated final weights mean that weighted totals for module respondents must not be interpreted as full-population counts.

The structural analysis remained at the teacher level. The 863 teachers were nested in 352 schools, with an average of 2.45 mathematics teachers per school, but between-school variation in T4SESDEV was only 0.011. A substantive multilevel mediation model was therefore not estimated: the available design contains limited within-school replication and the study does not formulate school-level constructs or effects. Nesting was nevertheless retained in inference through the official design and the auxiliary school-cluster estimators. The small intraclass correlation is interpreted as a descriptive feature of the outcome, not as permission to ignore the sampling design (Table 1).

### 2.2. Measures

All focal variables are OECD-derived TALIS indices reported on a metric with an international mean of 10 and a standard deviation of 2. T4AUTCH measures perceived autonomy over curriculum implementation, teaching methods, assessment activities, and lesson design. The primary mediator, T4SELF, combines self-efficacy in student engagement (T4SEENG), instruction (T4SEINS), and classroom management (T4SECLS). T4COMSEL, used only in the parallel extension, measures comfort with the social and emotional aspects of teaching and is interpreted as an SEL-specific disposition. T4GROMST measures belief in the malleability of ability.

The outcome, T4SESDEV, records the reported frequency of working on students’ management of emotions, thoughts, or behaviour; empathy; healthy relationships; and caring and constructive choices (OECD, 2025a). Higher values indicate greater reported emphasis, not observed implementation or student competence. The primary covariate set included gender, ISCED level, teaching experience category, and class-size category; ISCED was omitted from level-specific models. A sensitivity specification added professional collaboration in lessons (T4COLES), exchange of information among teachers (T4EXINF), and perceived classroom disruption (T4CLSDIS) as plausible common correlates of self-efficacy and the outcome.

The adjustment set was intentionally limited to characteristics plausibly antecedent to both perceived autonomy and the downstream constructs. Gender and teaching experience precede current working conditions, while class size and ISCED level describe the assigned teaching context. The same set was entered in the mediator and outcome equations; ISCED was omitted from level-specific models. This choice does not establish exchangeability, but it avoids automatically controlling for contemporaneous variables that may be consequences of autonomy or capability beliefs and could introduce collider or over-adjustment bias.

Professional collaboration in lessons, exchange of information among teachers, and classroom disruption were treated differently. Each could be related to both capability beliefs and reported social–emotional emphasis, but their temporal position relative to the mediator cannot be established. They were therefore added together in an extended covariate sensitivity model rather than in the primary specification. Empathy with students was excluded because it overlaps with one component of T4SESDEV, and T4COMSEL was reserved for the parallel extension because it represents the SEL-specific disposition under study. Supplementary Table S9e shows how conditioning on such pathway-overlapping variables changes the estimate without presenting that specification as the preferred confounding control.

### 2.3. Analytic Strategy

The analysis proceeded in four stages. First, descriptive distributions and item-level measurement evidence were examined. Second, the primary weighted model tested H1–H4 by regressing T4SELF on T4AUTCH and the covariates and regressing T4SESDEV on T4SELF, T4AUTCH, and the same covariates. The statistical indirect association was computed as a × b and the total association as c′ + ab. Third, the model was estimated separately within ISCED 2 and ISCED 3. Fourth, domain, parallel-disposition, subject-comparison, growth mindset, measurement error, and extended covariate analyses assessed robustness or scope; these were secondary to the confirmatory model.

Point estimates used TCHWGT-weighted ordinary least squares. Complete-case analysis was used, with *n* = 863 for the primary and domain models, *n* = 861 for the parallel and growth mindset models, and *n* = 860 for the extended covariate model. Re-estimation of the primary model on the stricter common extension sample produced no material change. Standardised coefficients were derived from weighted standard deviations.

Given the low focal-variable missingness and the 2.3% eligible-to-analytic reduction, complete-case analysis was considered acceptable for the primary model. As a composition check, the primary model was re-estimated on 856 teachers with complete data on every extension variable. The resulting a, b, and c′ paths were 0.383, 0.362, and 0.230, with an indirect association of 0.139, indicating that the small sample differences did not account for the focal result.

The proportion mediated is not reported. In a cross-sectional model that does not identify a mediated causal effect, dividing the indirect component by the total association would invite a mechanistic interpretation that the design cannot support. The ratio can also be unstable when the total association is imprecise or changes sign. The analysis therefore reports the indirect, direct, and total associations separately, each with design-based uncertainty.

### 2.4. Design-Based Inference

Level-specific standard errors and confidence intervals used the 100 official TALIS balanced repeated replication (BRR) replicate weights with Fay’s adjustment (k = 0.50; Rust & Rao, 1996). Because the replicate systems were constructed separately for ISCED 2 and ISCED 3, pooled-union variance was obtained by perturbing each population with its own replicate system while holding the other at its final weights and then summing the two variance contributions. Nonlinear quantities, including indirect associations and between-group differences, were recomputed within each perturbation. Full construction details and comparisons with school-clustered and school-bootstrap estimators are provided in Supplementary Table S8.

This design preserves each target population’s official replicate system without imposing an arbitrary pairing across levels. The resulting pooled intervals are design-based estimates for the investigator-defined union, not official TALIS population estimates.

Two auxiliary variance estimators were retained to assess the practical consequences of the design construction: school-clustered HC1 covariance estimation and a 5000-repetition school-cluster bootstrap in which complete schools were sampled with replacement (seed 20260814). Neither reproduces the stratification and separate calibration of the TALIS samples, so neither replaces Fay-BRR inference. Agreement among the estimators is reported only as a robustness comparison in Supplementary Table S8.

### 2.5. Measurement Evidence and Secondary Analyses

An item-level audit evaluated the seven scales carrying inference. Ordinal CFA used WLSMV and TCHWGT; a clustered robust-maximum-likelihood model provided a continuous-item sensitivity analysis. Reliability was estimated with categorical omega and school-cluster bootstrap intervals. ISCED comparability was examined with equal-threshold and equal-loading ordinal models and clustered configural, metric, and scalar sensitivity models. Sparse-category adjustments were limited to the ordinal audit; official OECD scale scores remained unchanged. Full item mappings, category diagnostics, fit indices, reliability estimates, discriminant evidence, and invariance results appear in Supplementary Tables S2–S4.

The five-factor primary measurement model distinguished instructional autonomy, the three efficacy domains, and T4SESDEV. It was compared with alternatives that collapsed the efficacy domains or all constructs. The seven-factor extension added T4COMSEL and T4GROMST, while an auxiliary twelve-item model was used only to obtain a reliability parameter corresponding to the complete item set underlying the OECD T4SELF composite. T4SELF remained an OECD composite of distinguishable efficacy domains rather than being redefined as a unidimensional latent factor.

Because T4COMSEL and T4SESDEV have content proximity and the same reporter, their separability was examined using disattenuated latent correlations, average variance extracted, heterotrait–monotrait ratios, and a direct comparison of the seven-factor model with an alternative that combined their items. Item mappings were checked against the OECD scale scores. Sparse-category adjustments were restricted to the ordinal audit: TT4G27I was excluded from the ordinal classroom-management factor, and the two lowest categories of TT4G61D were combined. Official scale scores and continuous-item sensitivity models retained the original values.

Ordinal invariance analyses compared equal-threshold and equal-loading models across ISCED levels; clustered robust-maximum-likelihood configural, metric, and scalar models served as continuous-item sensitivity analyses. School clustering could not be incorporated into the categorical WLSMV models in the software used. Consequently, the audit supplies evidence on dimensional ordering, reliability, and cross-level comparability, but it is not a structural estimation model and does not convert the observed-score comparisons into latent mean comparisons.

Secondary analyses decomposed T4SELF into its three domains, compared ISCED levels, added T4COMSEL in parallel, compared mathematics with other-subject teachers, examined growth mindset moderation, evaluated manifest-score measurement error following the sensitivity logic described by Cole and Preacher (2014), and added the extended covariate set. Detailed coefficients are reported in Supplementary Tables S5–S9. These analyses test robustness or contextual scope and do not alter the confirmatory status of H1–H4.

Dependent comparisons were recomputed within each replicate. Pairwise differences among the three efficacy-domain indirect associations were formed on the same teachers; ISCED path differences were estimated with all focal predictors and covariates interacted so that pooled differences reconciled with the separately fitted group models. The subject comparison used the same full-interaction principle. In the growth mindset model, T4AUTCH and T4GROMST were centred on their weighted means, and conditional indirect associations were evaluated at the mean and at one weighted standard deviation above and below it.

Measurement error analyses were presented as sensitivity analyses rather than corrected estimates. Random error in the mediator is expected to attenuate b and inflate c′, whereas error in the predictor can change these paths in the opposite direction (Cole & Preacher, 2014). The analysis therefore considered mediator-only and joint predictor–mediator reliability assumptions. Reliability parameters were treated as fixed, so the reported intervals reflect uncertainty in the structural estimates but not estimation error in omega.

### 2.6. Inferential Scope, Software, and Reproducibility

The model estimates associations and a statistical indirect association. Cross-sectional measurement cannot establish temporal ordering; shared teacher reporting may inflate, especially the b path; and unmeasured common causes remain possible. Structural and measurement error analyses were conducted in Python 3.12 using NumPy 2.5.2, pandas 3.0.0, and statsmodels 0.14.6. Item-level models, omega, and invariance analyses were conducted in R 4.3.3 using lavaan 0.6.17 and semTools 0.5.6. Stochastic school-cluster bootstrap procedures used seed 20260814; BRR inference uses fixed replicate weights. Code and variable-construction scripts are available from the corresponding author on reasonable request.

## 3. Results

### 3.1. Descriptive and Measurement Evidence

Weighted descriptive statistics are shown in Table 2. T4SESDEV was close to symmetric (skewness = 0.09), with no indication of a ceiling effect. Weighted correlations among the focal scales are reported in Supplementary Figure S1; T4AUTCH correlated 0.38 with T4SELF and 0.38 with T4SESDEV, while T4SELF correlated 0.47 with T4SESDEV. School intraclass correlations were 0.011 for T4SESDEV, 0.067 for T4SELF, and approximately zero for T4AUTCH.

The intended five-factor ordinal model outperformed alternatives that collapsed the efficacy domains or all constructs. Scaled fit was strong (CFI = 0.984, TLI = 0.980, RMSEA = 0.061, SRMR = 0.043), whereas the more conservative robust correction yielded mixed absolute fit; interpretation therefore relied on comparative ordering, substantial loadings, and reliability rather than on a single fit threshold. Categorical omega ranged from 0.821 to 0.920 for the primary component scales and was 0.964 for the full T4SELF item set. ISCED threshold and loading constraints produced absolute ΔCFI values below 0.010. T4COMSEL and T4SESDEV were empirically distinguishable in the seven-factor extension. Complete results are provided in Supplementary Tables S2–S4.

The three-factor alternative that collapsed the efficacy domains fitted less well (CFI = 0.975, RMSEA = 0.074, SRMR = 0.053), and the one-factor model was clearly inadequate (CFI = 0.841, RMSEA = 0.185, SRMR = 0.187); all standardised loadings in the intended model were at least 0.719. Robust corrections were more conservative than the scaled indices, especially for absolute fit. The measurement conclusion therefore rests on consistent comparative ordering, substantial loadings, and reliability evidence rather than on declaring exact fit from one index family.

The seven-factor extension provided more specific evidence for separating T4COMSEL from T4SESDEV. Their disattenuated correlation was 0.543, their heterotrait–monotrait ratio was 0.521, and constraining their items to one factor substantially worsened fit. Together with the invariance results summarised above, these findings support comparable observed-score use while remaining subject to the clustered-WLSMV limitation described in Section 2.5.

### 3.2. Primary Hypothesis Tests

All four primary hypotheses were supported. Table 3 links each primary hypothesis to its focal estimate, supporting analysis, and decision. The two secondary extensions are reported in Section 3.4. Perceived instructional autonomy was positively associated with overall teacher self-efficacy, B = 0.386, 95% CI [0.302, 0.471] (H1). Self-efficacy was positively associated with reported social–emotional emphasis after adjustment for autonomy and the covariates, B = 0.361, 95% CI [0.294, 0.429] (H2). The direct association remained positive, B = 0.229, 95% CI [0.156, 0.303] (H3).

The statistical indirect association through overall self-efficacy was B = 0.140, 95% CI [0.100, 0.179], β = 0.143 (H4), and the total association was B = 0.369, 95% CI [0.296, 0.442]. The mediator and outcome equations explained 17.0% and 28.4% of their respective variances. Because temporal ordering and causal identification are absent, the pattern is not interpreted as causal partial mediation.

Pooled-union design-based standard errors were close to the auxiliary school-clustered estimates: the BRR-to-clustered SE ratios were 1.07 for a, 0.96 for b, and 1.05 for c′. The Fay-BRR interval for the indirect association, [0.100, 0.179], was likewise close to the 5000-repetition school-cluster bootstrap interval, [0.102, 0.179]. This agreement indicates that the central inference was not an artefact of one variance estimator in this sample, while the Fay-BRR result remains primary because it preserves the official population-specific replicate systems.

### 3.3. Educational-Level Results

The indirect association was positive in both TALIS target populations: B = 0.153, 95% CI [0.095, 0.210] at ISCED 2 and B = 0.110, 95% CI [0.059, 0.160] at ISCED 3. Their difference was not statistically significant, B = −0.043, 95% CI [−0.120, 0.034].

The expression “offsetting patterns” refers to opposing point-estimate patterns in the component paths, not to established compensation between levels. The autonomy-to-self-efficacy and direct paths were larger at ISCED 2, whereas the self-efficacy-to-outcome point estimate was larger at ISCED 3. Because the latter difference was not statistically reliable, these results describe a level-specific configuration and generate a hypothesis for better-powered longitudinal designs; they do not establish distinct mechanisms.

At the path level, the autonomy-to-self-efficacy coefficient was 0.499 at ISCED 2 and 0.264 at ISCED 3, difference = −0.235, 95% CI [−0.396, −0.075]. The direct coefficients were 0.306 and 0.154, difference = −0.152, 95% CI [−0.297, −0.007]. By contrast, the self-efficacy-to-outcome coefficients were 0.306 and 0.416, difference = 0.109, 95% CI [−0.028, 0.247]. Thus, only two component-path differences were statistically reliable, and the apparently compensating b-path pattern remains descriptive (Table 4).

### 3.4. Robustness and Contextual Comparisons

The primary pattern did not depend on a single efficacy domain: indirect associations were positive for engagement (B = 0.111), instruction (B = 0.117), and classroom management (B = 0.095), with no statistically reliable pairwise differences. In the parallel extension (*n* = 861), overall self-efficacy and the SEL-specific disposition each retained a unique positive association with T4SESDEV and carried positive indirect associations of 0.102 and 0.077, respectively; their difference was not reliable. The 0.001 difference between the primary autonomy-to-self-efficacy coefficient (0.386; *n* = 863) and the parallel-model coefficient (0.387; *n* = 861) reflects the two-case sample difference rather than inconsistent rounding. H5 was therefore supported in both of its components, and the full parameter set appears in Supplementary Table S9c.

In the parallel model, the unique outcome associations were B = 0.264, 95% CI [0.196, 0.333] for overall self-efficacy and B = 0.237, 95% CI [0.169, 0.306] for T4COMSEL. Adding the SEL-specific disposition increased outcome R^2^ from 0.285 to 0.328 on the same 861-teacher sample, an increment of 0.044 computed from unrounded values, 95% CI [0.018, 0.069]. These results indicate nonredundancy in the fitted model, not a causal mechanism or a direct test of efficacy specificity.

The subject comparison included 6305 teachers, of whom 863 taught mathematics. No focal coefficient differed reliably between mathematics and other-subject teachers; the indirect associations were 0.140 and 0.135, difference = 0.005, 95% CI [−0.037, 0.047]. This null difference does not establish equivalence, but it supports framing mathematics as a substantively meaningful context rather than claiming a mathematics-specific statistical relationship. Full subject-comparison estimates appear in Supplementary Table S6.

Growth mindset did not reliably moderate the autonomy-to-efficacy path (B = 0.034, 95% CI [−0.014, 0.082]), so RQ1 is answered in the negative, with an index of moderated mediation of 0.012, 95% CI [−0.005, 0.030] (Table S7 and Figure S2). Measurement error sensitivity estimates placed the indirect association between 0.146 and 0.166 under the observed reliability specifications, compared with 0.140 in the manifest-score model (Table S5). Adding collaboration, information exchange, and classroom disruption reduced the same-sample indirect association by 33.8% to 0.092, 95% CI [0.058, 0.127], while it remained positive. These findings are treated as robustness evidence, not as additional primary claims; full estimates are reported in Supplementary Tables S5–S9.

The measurement error analyses behaved in the expected directions. At the estimated T4SELF reliability (ω = 0.964), the mediator-only indirect association was 0.146; jointly using the estimated T4AUTCH and T4SELF reliabilities produced 0.166, 95% CI [0.118, 0.214]. The unadjusted indirect estimate was therefore smaller than every reliability-adjusted sensitivity estimate, although this conclusion depends on classical-error assumptions. The direct association was not uniformly conservative because predictor and mediator error moved it in opposite directions.

In the extended covariate model, the a and b paths fell to 0.288 and 0.321, while c′ remained 0.219. The reduction in the indirect association indicates that collegial and classroom-climate conditions account for a meaningful portion of the observed pattern. Its remaining positive interval does not eliminate residual confounding, and the more attenuated pathway-overlap specifications in S9e are not interpreted as preferred adjusted estimates.

## 4. Discussion

### 4.1. Principal Finding and Educational Meaning

The central finding is straightforward: among mathematics teachers in the analysed TALIS 2024 Türkiye sample, perceived instructional autonomy, overall teacher self-efficacy, and reported emphasis on students’ social and emotional skill development were positively interconnected. Self-efficacy statistically carried part of the autonomy–reported emphasis association, while autonomy retained a positive direct association. The finding concerns a stable association pattern, not evidence that increasing autonomy would cause stronger efficacy beliefs or greater social–emotional attention.

Educationally, the result suggests that the discretion teachers perceive and their confidence in engaging students, organising instruction, and managing classrooms are relevant correlates of whether they report attending to social–emotional dimensions within mathematics teaching. That attention may appear through persistence during difficult tasks, constructive responses to error, regulation of uncertainty, respectful mathematical disagreement, and collaborative reasoning. The broader areas of empathy, healthy relationships, and caring choices may also enter through interaction norms, but T4SESDEV does not show how these emphases were enacted or whether students experienced them.

Four features delimit the substantive contribution. The primary paths and indirect association were positive; the pattern appeared across all three efficacy domains; the indirect association was present in both TALIS target populations; and broad self-efficacy remained relevant when an SEL-specific disposition was modelled in parallel. These convergences make the association pattern less dependent on a single operational choice. They do not, however, supply temporal order, independent observation, or causal identification. Robustness across specifications should therefore be read as stability of an observed pattern rather than as cumulative proof of a mechanism.

### 4.2. Autonomy, Capability, and the Limits of Reported Practice

The results are consistent with treating autonomy as a professional condition and self-efficacy as a capability belief. Autonomy describes the instructional options teachers perceive as available; capability beliefs are associated with whether teachers report taking up activities that require engagement, organisation, and classroom management. The positive direct path indicates that the two constructs are distinguishable correlates rather than interchangeable resources.

This interpretation connects more closely to Marks and Printy (2003) and Leithwood and Jantzi (2006) when their work is read as evidence that organisational conditions may be related to teaching through professional capacity rather than as direct antecedents of student outcomes. The present teacher-level pattern is conceptually compatible with that proposition, but it neither models leadership nor observes instructional quality. Leithwood and Jantzi’s distinction between reported practice and student achievement, together with Jerrim et al.’s (2023) weak and inconsistent autonomy associations with student mathematics outcomes, underscores that reported instructional emphasis remains several steps removed from student learning.

This boundary is substantive, not merely terminological. T4SESDEV is positioned early in any putative chain from working conditions to student learning and is reported by teachers rather than observed in classrooms. The appropriate inference is therefore that autonomy and capability beliefs covary with what teachers say they emphasise; the study offers no evidence that either variable improves achievement.

### 4.3. Mathematics as a Meaningful, Not Statistically Distinctive, Context

The mathematics focus is supported as contextual rather than comparative. Productive struggle, mathematical resilience, sociomathematical norms, argumentation, and collaborative problem solving make social and emotional processes pedagogically consequential in mathematics. However, the subject comparison detected no statistically reliable differences in the focal paths or indirect associations. The study therefore shows that the proposed structure is relevant within mathematics teaching, not that it is unique to mathematics teachers.

The absence of a reliable subject difference should not be interpreted as equivalence. The confidence intervals allow nontrivial differences, and coefficient similarity does not imply that empathy, relationships, emotional regulation, or constructive choices have identical instructional meanings across subjects. Future comparative work should use subject-responsive measures and designs developed specifically to test such differences.

The secondary analyses strengthen the scope of the primary result without changing its meaning. The association was positive across all three self-efficacy domains, both ISCED populations, and overall self-efficacy remained relevant alongside an SEL-specific disposition. The level-specific coefficients moved partly in opposing directions, but only two component-path differences were reliable. This pattern should be treated as a hypothesis for future research rather than as evidence of separate lower- and upper-secondary mechanisms.

The parallel extension adds a conceptually useful distinction. Overall self-efficacy describes perceived capability across engagement, instruction, and classroom management; T4COMSEL describes comfort with social–emotional content. Their independent coefficients are consistent with different constraints on reported practice. A teacher may be comfortable discussing social–emotional issues yet lack confidence in organising the class, while another may feel generally capable but be less willing to foreground such content. The fitted model is compatible with both dimensions mattering, although it cannot show that changing either would alter practice.

This interpretation depends on empirical separability. T4COMSEL and T4SESDEV were moderately related but did not behave as one factor, and T4SELF retained its unique association after T4COMSEL was entered. The result therefore addresses the concern that a broad efficacy composite merely proxies an unmeasured content-adjacent orientation. It remains bounded by shared reporting and by the fact that comfort is not a domain-matched efficacy belief; H5 should not be presented as a test of Bandura’s specificity principle.

The level-specific pattern also requires restraint. Similar indirect estimates at ISCED 2 and ISCED 3 arose alongside a stronger autonomy-to-efficacy path and direct path at ISCED 2, whereas the b-path point estimate was larger at ISCED 3. Only the first two differences were reliable, and the comparison was not preregistered. The configuration is consequently more useful as a hypothesis about how discretion and capability may be organised across educational levels than as evidence of two distinct mechanisms.

### 4.4. Limitations and Research Agenda

The most consequential limitation is shared measurement. T4SELF and T4SESDEV were reported by the same teacher, in the same questionnaire, about the same target class. Common response tendencies may therefore inflate especially the b path and the indirect association, and the extended covariate analysis cannot resolve this problem. A related limitation is the cross-sectional design: the proposed ordering has no demonstrated temporal precedence, and reverse or reciprocal relationships remain plausible.

A second set of limitations concerns construct interpretation. T4SESDEV measures reported frequency of emphasis rather than enacted teaching or student social–emotional outcomes, and its domain-general content is not equally close to every feature of mathematics instruction. T4COMSEL measures comfort, not a domain-matched efficacy belief, so the parallel extension cannot adjudicate Bandura’s specificity principle. The ordinal measurement audit also could not incorporate school-clustered standard errors, although clustered continuous-item sensitivity models and all structural analyses addressed nesting.

Further limits concern population and multiplicity. The pooled result describes an investigator-defined union of two separately calibrated TALIS target populations, not an official OECD population; level-specific results therefore remain essential. The target-class module covered two questionnaire forms, and analytic weight sums do not estimate the full mathematics teacher population. Finally, the level, subject, parallel, moderation, measurement error, and extended covariate analyses were not preregistered or adjusted for multiplicity. Confirmatory weight rests on H1–H4.

Future research should address the first two limitations directly. A longitudinal panel could establish whether changes in perceived autonomy precede changes in self-efficacy and reported emphasis. Multi-informant designs could combine teacher reports with structured classroom observations, student reports, or artefacts of mathematical tasks and discourse. Better-powered cross-level and cross-subject studies could test whether the component-path patterns replicate, while subject-responsive measures could distinguish the social–emotional processes embedded in mathematical activity from broader school-wide SEL.

The robustness analyses refine rather than remove the uncertainty. Official replicate-weight and school-cluster estimators produced similar precision; the indirect association remained positive across efficacy domains, levels, reliability assumptions, and the extended covariate set. At the same time, additional collegial and classroom-climate variables reduced the estimate by about one third, and no model can rule out common response tendencies in T4SELF and T4SESDEV. The stable finding is therefore the presence of a positive association pattern across reasonable specifications, not the size of a causal mediated effect.

For teacher education, the findings suggest a question rather than a prescription: whether opportunities for instructional decision-making are most productive when teachers also have the capability beliefs and subject-sensitive pedagogical resources needed to use that discretion. Programmes addressing social–emotional processes in mathematics may need to connect emotion regulation and relationships with concrete practices such as managing productive struggle, orchestrating mathematical discussion, responding to error, and structuring collaborative reasoning. Evaluating such programmes would require longitudinal or experimental evidence and observation of enacted practice.

A subject-responsive measurement agenda is also needed. T4SESDEV offers a valuable domain-general indicator, but its four areas are not equally proximal to mathematical activity. Future studies could distinguish regulation during challenging tasks, responses to mathematical error, participation in argumentation, and collaborative problem solving from broader relational or prosocial aims. Such measures would permit stronger tests of whether the same structural coefficients have comparable instructional meaning across subjects and educational levels.

## 5. Conclusions

Among mathematics teachers in the analysed TALIS 2024 Türkiye sample, greater perceived instructional autonomy was associated with greater overall teacher self-efficacy and greater reported emphasis on students’ social and emotional skill development, with a positive statistical indirect association through self-efficacy. The result remained positive across the principal design and robustness checks, but mathematics functioned as a substantively meaningful context rather than a statistically distinctive one. Because all focal variables were measured cross-sectionally through teacher reports, the findings do not show that increasing autonomy or self-efficacy would change classroom practice or student outcomes. Longitudinal and multi-informant research is needed to test temporal ordering and observed instructional enactment.

The contribution is accordingly twofold: the study identifies a focused association structure while retaining the separate TALIS population and replicate-weight design, and it clarifies that broad professional capability remains relevant alongside an SEL-specific disposition. These conclusions concern teacher reports within the mathematics-teaching context and should be tested with temporally ordered, multi-informant, and subject-responsive measures.

## Figures and Tables

**Table 1:** Study constructs, roles, and analytic samples.

Component	TALIS 2024 Variable	Definition/Value	Role
Perceived instructional autonomy	T4AUTCH	Autonomy over flexible curriculum implementation, teaching methods, assessment activities, and lesson design	X
Overall teacher self-efficacy	T4SELF	Overall index formed from T4SEENG, T4SEINS, T4SECLS	M
Comfort with social and emotional aspects of teaching	T4COMSEL	SEL-specific disposition	Parallel mediator (H5)
Growth mindset	T4GROMST	Belief that ability is malleable	Moderator (RQ1)
Social and emotional skill-development emphasis	T4SESDEV	Reported frequency of developing emotion/behaviour management, empathy, healthy relationships, caring choices	Y
Pooled analytic sample	—	863 teachers in 352 schools; ΣTCHWGT = 51,448.9	Union-population analysis
ISCED 2 population	ISCED level = 2	468 teachers in 194 schools; ΣTCHWGT = 28,632.6 (55.7%)	Co-primary analysis
ISCED 3 population	ISCED level = 3	395 teachers in 158 schools; ΣTCHWGT = 22,816.3 (44.3%)	Co-primary analysis
Teacher weight	TCHWGT	TALIS teacher final weight	Sampling weight
School cluster	IDSCHOOL	School identifier	Cluster ID for auxiliary checks

Note. T4SELF is treated as an OECD composite score rather than as a single reflective latent factor. ISCED 2 and ISCED 3 are separate TALIS target populations with separately calibrated weights. X denotes the predictor (T4AUTCH), M the mediator (T4SELF), and Y the outcome (T4SESDEV), as shown in Figure 1.

**Table 2:** Weighted descriptive statistics (*n* = 863).

Variable	*M*	*SD*	Min	Max	Skewness
T4AUTCH (instructional autonomy)	11.915	1.894	5.61	15.17	0.214
T4SELF (overall self-efficacy)	9.701	1.981	1.02	13.05	−0.042
T4SEENG (efficacy: engagement)	12.170	1.902	4.84	14.96	−0.129
T4SEINS (efficacy: instruction)	12.644	1.919	4.92	15.59	−0.065
T4SECLS (efficacy: classroom management)	12.968	1.904	5.18	15.37	−0.274
T4SESDEV (social–emotional emphasis)	11.916	1.854	6.37	14.94	0.092
T4COMSEL (comfort with social and emotional aspects)	12.171	1.910	5.83	15.02	−0.018
T4GROMST (growth mindset)	10.589	1.884	6.03	14.26	−0.019

Note. Statistics are weighted by TCHWGT. Scales are OECD-derived TALIS 2024 indices on a metric with an international mean of 10 and a standard deviation of 2. Intraclass correlations across schools: T4SESDEV = 0.011; T4SELF = 0.067; T4AUTCH ≈ 0.00.

**Table 3:** Summary of primary hypothesis tests, supporting analyses, and decisions.

Hypothesis	Focal Test and Estimate	Supporting Analysis	Decision
H1	T4AUTCH → T4SELF B = 0.386; SE = 0.043; β = 0.369; 95% CI [0.302, 0.471]	Primary weighted model, Section 3.2 (*n* = 863)	Supported
H2	T4SELF → T4SESDEV B = 0.361; SE = 0.034; β = 0.386; 95% CI [0.294, 0.429]	Primary weighted model, Section 3.2 (*n* = 863)	Supported
H3	T4AUTCH → T4SESDEV (direct association) B = 0.229; SE = 0.038; β = 0.234; 95% CI [0.156, 0.303]	Primary weighted model, Section 3.2 (*n* = 863)	Supported
H4	Indirect association: a × b B = 0.140; SE = 0.020; β = 0.143; 95% CI [0.100, 0.179]	Primary model with pooled-union Fay-BRR inference, Section 3.2; robustness in Section 3.3 and Section 3.4, and Tables S5 and S9	Supported

Note. *n* = 863 teachers in 352 schools. Point estimates use TCHWGT. Pooled-union inference sums the separate ISCED 2 and ISCED 3 Fay-BRR variance contributions from their official 100-replicate systems. R^2^ = 0.170, 95% CI [0.107, 0.233] for the mediator equation and 0.284, 95% CI [0.227, 0.342] for the outcome equation. All *p* < 0.001. H5 and RQ1 are secondary extensions reported in Section 3.4 and Supplementary Tables S7 and S9c.

**Table 4:** Level-specific paths and between-level differences.

Path	ISCED 2 B [95% CI]	ISCED 3 B [95% CI]	Difference (ISCED 3 − ISCED 2) [95% CI]
a: T4AUTCH → T4SELF	0.499 [0.379, 0.619]	0.264 [0.156, 0.371]	−0.235 [−0.396, −0.075]
b: T4SELF → T4SESDEV	0.306 [0.216, 0.396]	0.416 [0.311, 0.520]	0.109 [−0.028, 0.247]
c′: T4AUTCH → T4SESDEV	0.306 [0.203, 0.409]	0.154 [0.052, 0.256]	−0.152 [−0.297, −0.007]
Indirect: a × b	0.153 [0.095, 0.210]	0.110 [0.059, 0.160]	−0.043 [−0.120, 0.034]

Note. Group models retained gender, teaching experience, and class size as covariates (*n* = 468 and 395). Each level-specific interval uses that population’s official 100 Fay-BRR replicate weights. Difference variances sum the two level-specific BRR contributions. Differences are computed from unrounded coefficients and may therefore differ in the third decimal from differences taken between the rounded values shown.

## Data Availability

The TALIS 2024 public-use data files analysed in this study are publicly available from the OECD at https://www.oecd.org/education/talis/ (accessed on 12 August 2026). The data were not generated by the authors and are not redistributed here. The analysis code, the variable-construction script, and all Supplementary Tables are available from the corresponding author on reasonable request.

## Supplementary Materials

The following supporting information can be downloaded at https://www.mdpi.com/article/10.3390/bs16091601/s1, Table S1: level-specific weighted descriptive statistics and listwise versus available-case comparison; Table S2: item-level response category frequencies and ordinal adjustments; Table S3: measurement models, reliability, and invariance; Table S4: extended measurement model and discriminant evidence; Table S5: measurement error sensitivity; Table S6: subject comparison; Table S7: growth mindset moderation; Table S8: design effects, variance estimators, and pooled-union BRR construction; Table S9: additional domain, ISCED, parallel-model, extended covariate, and adjustment-set sensitivity results; Figure S1: weighted correlation matrix; Figure S2: conditional indirect association across growth mindset.
