# Supplementary material for: Instructional Autonomy, Teacher Self-Efficacy, and Reported Emphasis on Students’ Social and Emotional Skills Development: A Design-Sensitive Analysis of Mathematics Teachers in TALIS 2024 Türkiye
Source: Behav Sci (Basel). 2026 Sep 9;16(9):1601. doi: 10.3390/bs16091601 (PMC13603465; doi:10.3390/bs16091601)
Supplement: Supplementary file 1 [file behavsci-16-01601-s001.zip › behavsci-4539159-supplementary.pdf]

Supplementary Materials

Instructional Autonomy, Teacher Self-Efficacy, and Reported Emphasis on Students' Social and Emotional Skill Development: A Design-Sensitive Analysis of Mathematics Teachers in TALIS 2024 Türkiye

Ramazan Erol and Atilla Özdemir

Table S1. Level-specific weighted descriptive statistics, and listwise versus available-case comparison

S1a. By educational level

Variable	ISCED 2 *M*	ISCED 2 *SD*	ISCED 3 *M*	ISCED 3 *SD*
T4AUTCH	11.987	1.837	11.825	1.960
T4SELF	9.900	2.050	9.452	1.861
T4SEENG	12.142	1.994	12.205	1.777
T4SEINS	12.680	1.947	12.599	1.883
T4SECLS	12.886	1.983	13.071	1.795
T4SESDEV	12.023	1.871	11.781	1.823
T4COMSEL	12.124	1.979	12.230	1.817
T4GROMST	10.528	1.817	10.666	1.962

Note. For the five primary scale scores, n = 468 at ISCED 2 and 395 at ISCED 3. T4COMSEL was available for 468 / 393 teachers and T4GROMST for 467 / 394 teachers at ISCED 2 / 3, respectively. Statistics are weighted by TCHWGT. The target-class module was carried by questionnaire forms 2 and 3, which represented 66.5% of the summed teacher weight at ISCED 2 and 66.4% at ISCED 3. After item-level availability of the target-class subject designation, valid subject data represented 65.5% and 64.6% of the summed teacher weight, respectively. Because form assignment was random and the coverage rates were nearly identical across levels, these percentages provide no descriptive indication of marked differential form coverage between the two ISCED populations.

S1b. Listwise analytic sample versus available-case estimates

Variable	Listwise *M*	Listwise *SD*	Available-case *M*	Available-case *SD*	Difference in *M*
T4AUTCH	11.915	1.894	11.934	1.900	-0.019
T4SELF	9.701	1.981	9.699	1.980	+0.002
T4SEENG	12.170	1.902	12.176	1.898	-0.006
T4SEINS	12.644	1.919	12.642	1.914	+0.002
T4SECLS	12.968	1.904	12.970	1.900	-0.002
T4SESDEV	11.916	1.854	11.924	1.862	-0.009
T4COMSEL	12.171	1.910	12.156	1.914	+0.015
T4GROMST	10.589	1.884	10.590	1.878	-0.001

Note. Listwise N = 863. Available-case n by variable was 874 (T4AUTCH), 876 (T4SELF), 879 (T4SEENG), 876 (T4SEINS), 877 (T4SECLS), 875 (T4SESDEV), 879

(T4COMSEL), and 880 (T4GROMST). The largest absolute difference in weighted means is 0.019 scale-score units, on a metric with an international standard deviation of 2; listwise deletion therefore did not materially shift the analytic sample.

Table S2. Item-level response category frequencies by ISCED group

Scale	Item	Cat 1 (2/3)	Cat 2 (2/3)	Cat 3 (2/3)	Cat 4 (2/3)
T4AUTCH (instructional autonomy)	TT4G57A	11 / 19	98 / 90	259 / 206	99 / 78
	TT4G57B	2 / 2	38 / 56	298 / 215	130 / 118
	TT4G57C	9 / 9	65 / 74	263 / 208	126 / 104
	TT4G57E	3 / 5	47 / 46	255 / 201	157 / 138
T4SEENG (efficacy: student engagement)	TT4G27A	1 / 1	55 / 29	239 / 230	172 / 132
	TT4G27B	3 / 3	73 / 39	223 / 215	167 / 136
	TT4G27E	6 / 4	108 / 75	225 / 203	128 / 112
	TT4G27G	3 / 3	61 / 35	235 / 204	166 / 150
T4SEINS (efficacy: instruction)	TT4G27C	5 / 3	58 / 40	249 / 203	156 / 148
	TT4G27J	3 / 1	77 / 78	246 / 192	141 / 122
	TT4G27K	2 / 2	23 / 15	205 / 178	236 / 199
	TT4G27L	3 / 3	69 / 72	232 / 206	161 / 112
T4SECLS (efficacy: classroom management)	TT4G27D	2 / 2	52 / 22	233 / 196	179 / 173
	TT4G27F	2 / 1	19 / 15	191 / 180	254 / 197
	TT4G27H	2 / 2	33 / 20	236 / 197	196 / 174
	TT4G27I	4 / 0 †	52 / 36	236 / 195	176 / 161
T4SESDEV (social-emotional emphasis)	TT4G61B	3 / 3	74 / 84	281 / 237	107 / 69
	TT4G61D	0 / 3 †	58 / 53	268 / 236	139 / 100
	TT4G61E	1 / 0 †	57 / 54	276 / 232	134 / 105
	TT4G61F	2 / 2	68 / 76	277 / 223	118 / 86

Note. Cells give the unweighted count in ISCED 2 / ISCED 3. Higher categories indicate greater autonomy, efficacy, or frequency. † marks a category that is empty in one group.

Ordinal adjustments applied

Two adjustments were applied in the main ordinal measurement models, with one additional category-collapsing step used only for the multi-group invariance models. Original item values were retained for all analyses using official OECD scale scores and for the continuous-item sensitivity models.

- **TT4G27I**, category 1: n = 4 in ISCED 2 and 0 in ISCED 3.
- **TT4G61D**, category 1: n = 0 in ISCED 2 and 3 in ISCED 3.

- **TT4G61E**, category 1: n = 1 in ISCED 2 and 0 in ISCED 3.

1. **TT4G27I** was excluded from the ordinal classroom-management factor because its lowest category had no support in ISCED 3, so its extreme-category thresholds were not jointly estimable across groups. Retaining it changes the five-factor solution negligibly (20-item model: CFI = .983, RMSEA = .062, versus 19-item model: CFI = .984, RMSEA = .061) and raises the categorical omega of the classroom-management scale from .821 to .863.
2. **TT4G61D**: the two lowest categories were combined because the lowest category was empty in ISCED 2.
3. In the multi-group invariance models only, the two lowest categories of all four T4SESDEV items were combined, because **TT4G61E** additionally has an empty lowest category in ISCED 3.

Table S3. Estimated measurement models

Model	Estimator	Items	χ^2	df	CFI (scaled / robust)	TLI (scaled / robust)	RMSEA (scaled / robust)	SRMR
Five-factor (target)	WLSMV	19	568.49	142	.984 / .911	.980 / .893	.061 / .106	.043
Three-factor (efficacy collapsed)	WLSMV	19	805.30	149	.975 / .863	.971 / .843	.074 / .128	.053
One-factor	WLSMV	19	4271.79	152	.841 / .457	.821 / .389	.185 / .252	.187
Five-factor, TT4G27I retained	WLSMV	20	641.10	160	.983 / .912	.980 / .896	.062 / .104	.044
T4SELF reliability model (11 items)	WLSMV	11	—	—	—	—	—	—
T4SELF reliability model (12 items)	WLSMV	12	—	—	—	—	—	—
Five-factor, continuous-item MLR	MLR	19	—	—	.959	.951	.056	.043

Note. All ordinal models used TCHWGT. School clustering could not be incorporated in the categorical estimator (lavaan 0.6.17) and was addressed in the MLR sensitivity model and in all structural analyses. Scaled and robust approximate fit indices are reported side by side because they differ substantially under mean- and variance-adjusted estimation. The two T4SELF rows are auxiliary reliability models only; they are not used as evidence that the broad T4SELF composite is unidimensional.

Categorical omega (Green–Yang) with 95% school-cluster bootstrap confidence intervals

Scale	Items	ω	95% CI
T4AUTCH	4	0.834	[0.815, 0.856]
T4SEENG	4	0.920	[0.889, 0.943]
T4SEINS	4	0.823	[0.788, 0.848]
T4SECLS	3	0.821	[0.792, 0.856]

Scale	Items	ω	95% CI
T4SESDEV	4	0.895	[0.876, 0.915]
T4SELF, 11-item ordinal set	11	.950	[.941, .962]
T4SELF, full 12-item auxiliary set	12	.964	—

Note. Confidence intervals are based on 200 school-cluster bootstrap repetitions (seed 20260814); 197 repetitions converged. The full 12-item T4SELF value (.964) is a point estimate from the auxiliary reliability model and is used only as the measurement-error sensitivity anchor.

Scale-by-scale ISCED measurement invariance (ΔCFI)

Scale	Configural CFI	Δ CFI, equal thresholds	Δ CFI, equal thresholds + loadings
T4AUTCH	0.996	+0.0019	+0.0007
T4SEENG	0.998	+0.0003	+0.0005
T4SEINS	0.996	-0.0012	+0.0011
T4SECLS	1.000	-0.0002	-0.0001
T4SESDEV	0.999	-0.0001	+0.0001

Note. For ordinal indicators, thresholds carry the function that intercepts serve in the continuous case, so the two steps together constitute the ordinal analogue of scalar invariance. Every change is far inside the conventional −.010 criterion. Each single-factor model has three or four indicators and therefore very few degrees of freedom, so RMSEA is uninformative at this level and ΔCFI is used as the decision criterion.

Table S4. Extended measurement model including the two extension constructs

Seven-factor ordinal CFA (WLSMV, TCHWGT, listwise n = 784; 434 ISCED 2 and 350 ISCED 3; 25 items): $\chi^2(254) = 658.32$, CFI = .986 (robust .915), TLI = .984 (robust .900), RMSEA = .045 (robust .086), SRMR = .040. T4COMSEL and T4SESDEV were empirically separable: their latent correlation was .543, HTMT = .521, and AVE was .685 and .833, respectively. Collapsing their seven items onto one factor degraded fit substantially, $\chi^2(260) = 1265.96$, ΔCFI = −.021, ΔRMSEA = +.025, ΔSRMR = +.025; scaled $\Delta\chi^2(6) = 206.69$, $p < .001$.

Disattenuated latent correlations and average variance extracted (seven-factor solution)

**Factor**	**AVE**	**1**	**2**	**3**	**4**	**5**	**6**	**7**
1. T4AUTCH	.698	—						
2. T4SEENG	.770	.378	—					
3. T4SEINS	.645	.460	.849	—				
4. T4SECLS	.721	.411	.838	.869	—			
5. T4SESDEV	.833	.454	.536	.504	.448	—		
6. T4COMSEL	.685	.410	.536	.531	.522	.543	—	
7. T4GROMST	.755	−.019	−.065	−.020	−.003	−.045	−.070	—

Note. Correlations are between latent factors in the seven-factor ordinal model and are therefore disattenuated for measurement error. AVE = average variance extracted. For the pair of substantive interest, T4COMSEL and T4SESDEV, the squared correlation is .295, below the AVE of either factor, which satisfies the Fornell-Larcker criterion. The heterotrait-monotrait ratio for that pair is .521, against .890 to .898 among the three efficacy domains.

Categorical omega point estimates in the seven-factor listwise sample

**Scale**	**Items**	**ω (n = 784)**
T4AUTCH	4	.836
T4SEENG	4	.919
T4SEINS	4	.819
T4SECLS	3	.819
T4SESDEV	4	.894
T4COMSEL	3	.789
T4GROMST	3	.845
T4SELF, 11-item ordinal set	11	.950
T4SELF, full 12-item auxiliary set	12	.964

Reliability by educational level

Scale	ω at ISCED 2	ω at ISCED 3
T4AUTCH	.829	.836
T4SELF item set	.966	.931

ISCED invariance for the two extension constructs

Scale	Δ CFI, equal thresholds	Δ CFI, equal thresholds + loadings
T4COMSEL	−.0008	+.0007
T4GROMST	.0000	.0000

Note. The seven-factor CFA analysis N is the number of teachers with complete responses on all 25 model items. T4GROMST is a three-indicator factor, so the configural model is saturated within each group and the invariance test is limited to

the threshold and loading steps. The T4GROMST items (TT4G30A–C) are worded as fixed-mindset statements; the reverse-scored item mean correlates approximately .995 with the OECD T4GROMST score, confirming the scoring direction. The 12-item T4SELF estimate is reported as an auxiliary reliability parameter and does not imply a single-factor interpretation of the composite. The omega values above are common-sample point estimates from the 784 teachers with complete responses on all 25 items. Scale-specific school-cluster bootstrap confidence intervals for the primary component scales are reported with their matching point estimates in Table S3. A common-sample bootstrap was not run, so unmatched intervals are not displayed here.

Table S5. Measurement-error sensitivity under alternative reliability assumptions

Sensitivity specification	ω_X	ω_M	a	b	c′	ab
None	1.000	1.000	0.386	0.361	0.229	0.140
Mediator only (observed ω_M)	1.000	0.964	0.386	0.378	0.223	0.146
Predictor only (observed ω_X)	0.834	1.000	0.465	0.341	0.285	0.159
Both observed reliabilities	0.834	0.964	0.465	0.358	0.278	0.166

The sensitivity analysis was estimated on the primary analytic sample of 863 teachers, in which the unadjusted autonomy-to-self-efficacy coefficient is 0.386. The value 0.387 reported in S9c belongs to the 861-teacher parallel-model sample. Design-based 95% confidence intervals were obtained from the two official TALIS replicate-weight systems by perturbing one ISCED population at a time and summing the two Fay-BRR variance contributions; reliability coefficients were held fixed. Mediator only (ω_M = .964): a [.302, .471], b [.307, .449], c′ [.149, .297], ab [.104, .188]. Predictor only (ω_X = .834): a [.363, .567], b [.270, .412], c′ [.193, .377], ab [.113, .204]. Both observed reliabilities: a [.363, .567], b [.283, .432], c′ [.185, .370], ab [.118, .214].

Note. These are sensitivity analyses rather than identified corrections. Error in the mediator attenuates b and inflates c′, whereas error in the predictor attenuates a and c′ and inflates b. The indirect association is larger than the unadjusted estimate under every adjustment considered. Reliability coefficients are treated as fixed, so these intervals reflect sampling variability in the structural parameters but not uncertainty in the reliability estimates.

Table S6. Subject comparison on the full Türkiye teacher sample

Model re-estimated on all teachers at ISCED 2 and ISCED 3 with complete data on the focal variables (n = 6,305 in 429 schools; 863 mathematics, 5,442 other subjects). Subject was

identified by TT4G48A (primary subject category of the target class). The mathematics indicator was interacted with the focal predictors and with every covariate, so each difference equals the difference between separately estimated subject-group coefficients.

Path	Mathematics	Other subjects	Difference (math − other)	95% CI
a: T4AUTCH → T4SELF	0.386	0.417	−0.031	[−0.118, 0.057]
b: T4SELF → T4SESDEV	0.361	0.323	0.038	[−0.036, 0.112]
c′: T4AUTCH → T4SESDEV	0.229	0.272	−0.043	[−0.123, 0.038]
Indirect: a × b	0.140	0.135	0.005	[−0.037, 0.047]

Note. The subject comparison spans two separately calibrated TALIS target populations. For the pooled union, each ISCED population was perturbed separately with its own 100 Fay-BRR replicate weights while the other population remained at its full final weights; the two variance contributions were then summed. This avoids imposing an arbitrary pairing between replicate columns from separately constructed replicate systems. No statistically reliable subject differences were detected, but moderate differences cannot be excluded.

Subject distribution of the comparison sample

Subject	n	Subject	n
Reading, writing and literature	1,384	Technology	252
Mathematics	863	Arts	264
Science	705	Physical education	328
Social studies	586	Religion and/or ethics	341
Modern foreign languages	481	Practical and vocational skills	548
Ancient Greek and/or Latin	2	Other	551

Table S7. Growth mindset as a boundary condition

Estimated on the 861 teachers with complete data on T4GROMST. The predictor and the moderator were centred on their TCHWGT-weighted means. Design-based inference from the 100 TALIS replicate weights.

Parameter	*B*	*SE*	95% CI	*t*
a_1: T4AUTCH → T4SELF at mean growth mindset	0.380	0.043	[0.295, 0.464]	8.81
Growth mindset → T4SELF	0.047	0.036	[−0.024, 0.118]	1.29
a_3: T4AUTCH × growth mindset	0.034	0.024	[−0.014, 0.082]	1.39
b: T4SELF → T4SESDEV	0.360	0.034	[0.293, 0.427]	10.48
Index of moderated mediation	0.012	0.009	[−0.005, 0.030]	1.38

Conditional indirect associations were 0.114 at one weighted standard deviation below the mean of growth mindset, 0.137 at the mean, and 0.160 one standard deviation above.

Note. The disattenuated correlations between T4GROMST and every other factor in Table S4 range from −.070 to −.003, which anticipates this result.

Table S8. Design effects and the comparison of variance estimators

Reported for transparency about why school-cluster procedures approximated the design-based standard errors closely in this application.

Weight variation

Kish design effect from unequal weighting, $1 + CV^2(w) = 1.311$; effective sample size 659 against a nominal 863.

Design effects on the focal coefficients

Path	*SE* simple random	*SE* weighted	*SE* weighted + clustered	Weighting deff	Clustering deff	Total deff
a	0.0326	0.0329	0.0402	1.02	1.50	1.52
b	0.0294	0.0298	0.0358	1.03	1.45	1.48
c′	0.0301	0.0308	0.0358	1.05	1.35	1.41

Design-based against school-cluster standard errors

Path	*SE* (BRR)	*SE* (school-clustered)	Ratio
a	0.0429	0.0402	1.07
b	0.0345	0.0358	0.96
c′	0.0375	0.0358	1.05

Note. Unequal weighting has little effect on the focal regression coefficients, while clustering accounts for most of the difference from simple-random-sampling standard errors. For the investigator-defined pooled union, the design-based standard errors shown here sum the separate ISCED 2 and ISCED 3 Fay-BRR variance contributions

rather than pairing replicate columns across populations. The cluster-based estimates are reported only as an auxiliary comparison.

Pooled-union Fay-BRR construction

ISCED 2 and ISCED 3 have separately calibrated final weights and separately constructed 100-replicate Fay-BRR systems. Replicate columns were therefore not paired across populations. For each ISCED 2 replicate, ISCED 2 weights were perturbed while ISCED 3 remained at its final weights; the procedure was then reversed for each ISCED 3 replicate. The two Fay-BRR variance contributions were summed. Nonlinear statistics, including $a \times b$ and coefficient differences, were recomputed under every perturbation before combination.

This procedure retains each official replicate system without treating the investigator-defined union as an OECD-calibrated population. Level-specific estimates use the official population-specific BRR procedure directly. School-clustered HC1 and paired school-cluster bootstrap intervals are provided only as design-approximating comparisons.

Table S9. Additional robustness and extension analyses

S9a. Self-efficacy domain decomposition

Specification	B	95% CI
Indirect via engagement self-efficacy	0.111	[0.076, 0.146]
Indirect via instruction self-efficacy	0.117	[0.080, 0.153]
Indirect via classroom-management self-efficacy	0.095	[0.063, 0.127]
Instruction − engagement	0.006	[−0.021, 0.032]
Instruction − classroom management	0.021	[−0.009, 0.052]
Engagement − classroom management	0.016	[−0.011, 0.043]

Note. Domain-specific models used the same 863 teachers and the same pooled-union Fay-BRR construction as the primary model. Pairwise differences were recomputed within every replicate.

S9b. ISCED-specific paths and differences

Path	ISCED 2 B [95% CI]	ISCED 3 B [95% CI]	Difference (ISCED 3 − ISCED 2) [95% CI]
a: T4AUTCH → T4SELF	0.499 [0.379, 0.619]	0.264 [0.156, 0.371]	−0.235 [−0.396, −0.075]
b: T4SELF → T4SESDEV	0.306 [0.216, 0.396]	0.416 [0.311, 0.520]	0.109 [−0.028, 0.247]
c′: T4AUTCH → T4SESDEV	0.306 [0.203, 0.409]	0.154 [0.052, 0.256]	−0.152 [−0.297, −0.007]
Indirect: a × b	0.153 [0.095, 0.210]	0.110 [0.059, 0.160]	−0.043 [−0.120, 0.034]

Note. Difference direction is ISCED 3 − ISCED 2. Difference variances sum the two population-specific Fay-BRR contributions.

S9c. Parallel model with overall self-efficacy and the SEL-specific disposition

Parameter	B	95% CI
a_1: T4AUTCH → T4SELF	0.387	[0.302, 0.471]
a_2: T4AUTCH → T4COMSEL	0.323	[0.237, 0.408]
Difference $a_1 - a_2$	0.064	[−0.016, 0.145]

Parameter	B	95% CI
b_1: T4SELF → T4SESDEV	0.264	[0.196, 0.333]
b_2: T4COMSEL → T4SESDEV	0.237	[0.169, 0.306]
c′: T4AUTCH → T4SESDEV	0.191	[0.121, 0.261]
Indirect via T4SELF	0.102	[0.067, 0.137]
Indirect via T4COMSEL	0.077	[0.048, 0.105]
Difference between indirect associations	0.026	[−0.021, 0.072]
Total indirect	0.179	—
Increment in R^2 over the single-mediator model	0.044	[0.018, 0.069]

Note. The parallel model used n = 861. The autonomy-to-self-efficacy coefficient is 0.387 in this sample, compared with 0.386 in the n = 863 primary sample; the difference reflects sample composition rather than inconsistent reporting.

S9d. Extended-covariate sensitivity

Parameter	Primary model on same sample	Extended specification	95% CI, extended
a: T4AUTCH → T4SELF	—	0.288	[0.210, 0.366]
b: T4SELF → T4SESDEV	—	0.321	[0.250, 0.392]
c′: T4AUTCH → T4SESDEV	—	0.219	[0.147, 0.291]
Indirect: a × b	0.140	0.092	[0.058, 0.127]

Note. n = 860. The extended specification added T4COLES, T4EXINF, and T4CLSDIS to both equations. The indirect association decreased by 33.8%. Empathy and T4COMSEL were not included as confounders because they overlap with or lie conceptually close to the pathway of interest.

S9e. Adjustment-set rationale, pathway-overlap sensitivity, and the proportion mediated

The four primary covariates were gender, ISCED level, teaching-experience category, and class-size category. They were treated as pre-exposure demographic or contextual characteristics plausibly related to perceived instructional autonomy and the downstream constructs. This selection reduces obvious adjustment for variables that could follow autonomy or capability beliefs, but it does not establish that the set is causally sufficient. Gender and teaching experience precede current working conditions; class size and ISCED level describe the assigned teaching context. Variables plausibly downstream, such as job satisfaction, were deliberately excluded.

Two further TALIS variables that might appear to belong in the adjustment set were excluded on conceptual grounds. Empathy with students (T4EMPS) overlaps in content with the outcome, one of whose four component items concerns developing the ability of students to empathise with others. Comfort with the social and emotional aspects of teaching (T4COMSEL) is modelled as a parallel SEL-specific disposition in S9c rather than as a confounder. Their consequences are nevertheless reported below for transparency.

**Specification**	**Indirect association (a × b)**
Primary adjustment set (Section 3.2)	0.140
Plus T4COLES, T4EXINF, and T4CLSDIS (S9d)	0.092
Plus empathy with students (T4EMPS)	0.044
Plus all five additional variables	0.021

Note. All specifications are estimated on the same teachers as the corresponding primary model. The final two rows show sensitivity to conditioning on variables that overlap with the outcome or may lie on the proposed pathway. Given the cross-sectional design, they are not treated as preferred confounder-adjusted estimates or as evidence that the focal association is spurious.

The proportion of the total association represented by the indirect component is not reported anywhere in the article. Under a cross-sectional design in which the identification conditions for a mediated effect are not met, this ratio cannot be interpreted as the share of an effect operating through the mediator, its sampling distribution is unstable, and reporting it would invite the mechanistic reading that the analysis is written to avoid.

Figure S1. Weighted correlation matrix of all focal scales

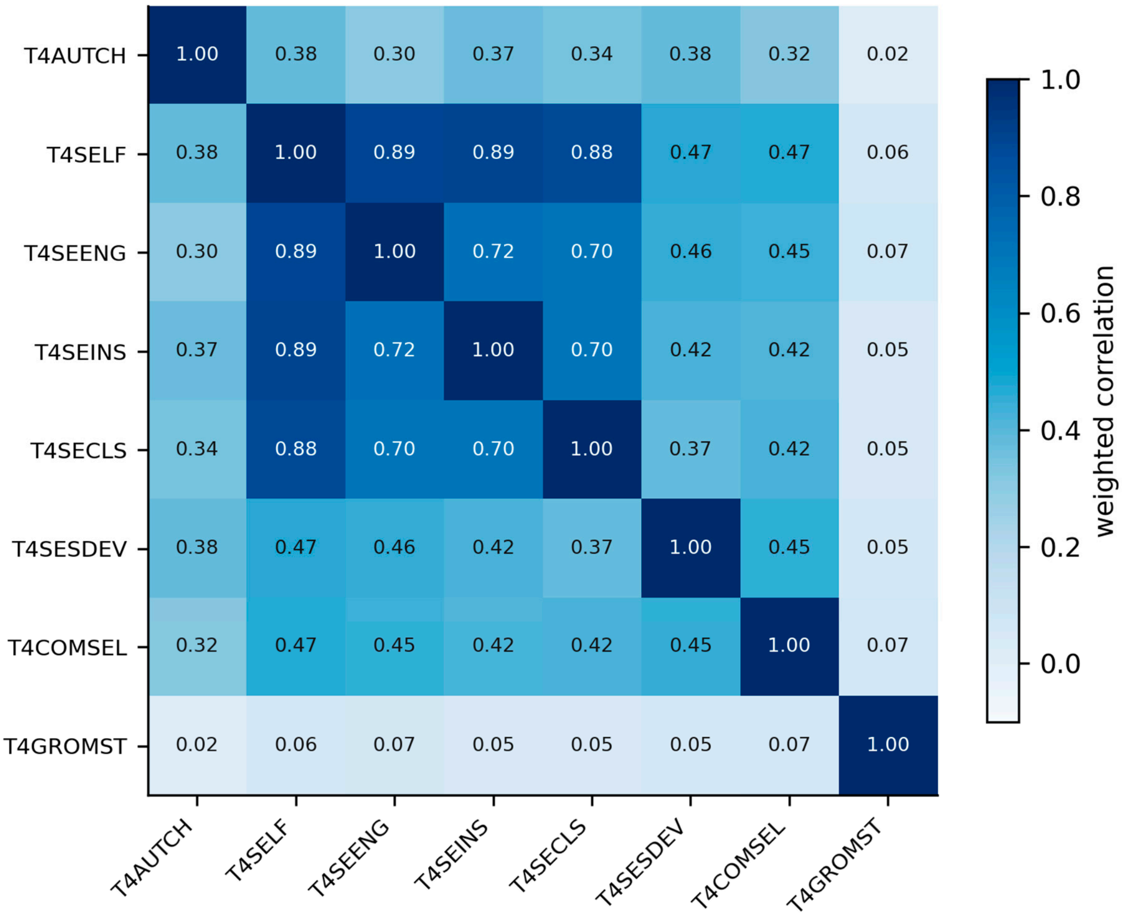

Note. Weighted by TCHWGT, n = 859 complete cases on all eight focal scale scores, including T4GROMST.

Figure S2. Conditional indirect association across levels of growth mindset

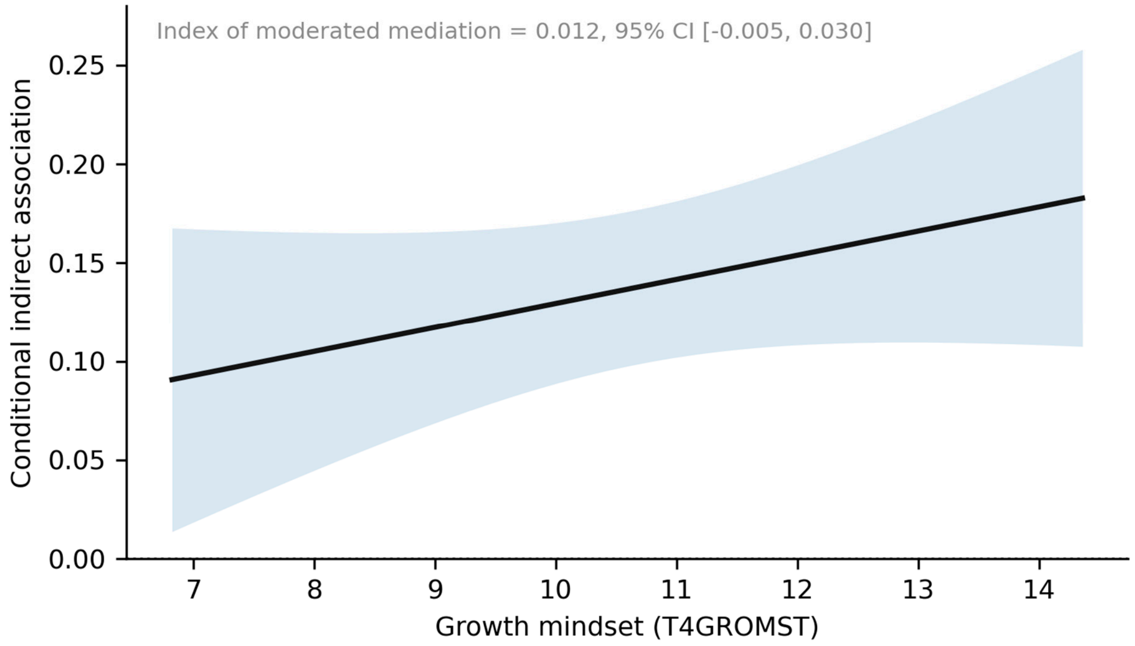

Note. The shaded band is a 95% pooled-union design-based interval obtained by summing the separate ISCED 2 and ISCED 3 Fay-BRR variance contributions. The index of moderated mediation was 0.012, 95% CI [−0.005, 0.030].

Software

Structural models and measurement-error sensitivity functions: Python 3.12 (NumPy, pandas, statsmodels). Item-level measurement models, categorical omega, and invariance tests: R 4.3.3 (lavaan 0.6.17, semTools 0.5.6). Level-specific structural inference uses the 100 official TALIS replicate weights with Fay's adjustment. For pooled-union quantities, the ISCED 2 and ISCED 3 replicate systems are applied separately and their variance contributions are summed. Stochastic school-cluster bootstrap procedures used seed 20260814; BRR inference itself uses fixed replicate weights and requires no seed.
